# Voltammetric Detection of Vanillylmandelic Acid and Homovanillic Acid Using Urea-Derivative-Modified Graphite Electrode

**DOI:** 10.3390/s23073727

**Published:** 2023-04-04

**Authors:** Tatiana V. Shishkanova, František Králík, Alla Synytsya

**Affiliations:** Department of Analytical Chemistry, University of Chemistry and Technology, Technická 5, 166 28 Prague, Czech Republic

**Keywords:** neuroblastoma, urea-derivative receptor, electrochemical impedance spectroscopy, differential pulse voltammetry

## Abstract

Vanillylmandelic acid (VMA) and homovanillic acid (HVA) are diagnostic markers of neuroblastoma. The purpose of this study was to understand the reason for the discrimination of structural analogues (VMA and HVA) onto a graphite electrode coated with an electrochemically oxidized urea derivative. Density functional theory calculations (DFT), FTIR spectroscopic measurements, and electrochemical impedance spectroscopic measurements were used in this work. Density functional theory calculations (DFT) were used to identify the most suitable binding sites of the urea derivative and to describe possible differences in its interaction with the studied analytes. The FTIR measurement indicated the enhancement and disappearance of NH vibrations on graphite and platinum surfaces, respectively, that could be connected to a different orientation and thus provide accessibility of the urea moiety for the discrimination of carboxylates. Additionally, the higher the basicity of the anion, the stronger the hydrogen-bonding interaction with –NH-groups of the urea moiety: VMA (pK_b_ = 10.6, K_Ads_ = (5.18 ± 1.95) × 10^5^) and HVA (pK_b_ = 9.6, K_Ads_ = (4.78 ± 1.58) × 10^4^). The differential pulse voltammetric method was applied to detect VMA and HVA as individual species and interferents. As individual analytes, both HVA and VMA can be detected at a concentration of 1.99 × 10^−5^ M (RSD ≤ 0.28, recovery 110–115%).

## 1. Introduction

**Figure 1:**
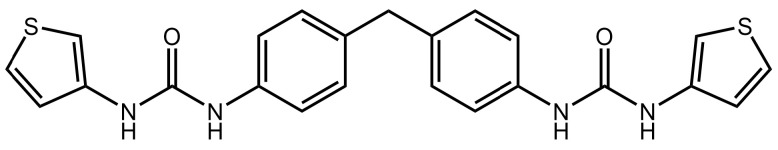
Chemical structure of the urea derivative used for the electrochemical modification of graphite electrode surfaces in this study.

The design of supramolecular receptors is a way to develop selective sensor devices that respond to specific analytes [1,2,3,4]. The deposition of a supramolecular receptor on a suitable electrode surface offers the possibility of preparing a selective electrode–solution interface [5]. An electrochemical sensor detects the interaction between an analyte of interest and the modified electrode through changes in the generated electrochemical signal (e.g., capacitance, charge-transfer resistance, current). Electrochemical techniques make it possible to both evaluate the affinity of the modified surface towards the analyte and obtain quantitative information. Metabolites of catecholamines, namely vanillylmandelic acid (VMA) and homovanillic acid (HVA), are important in the diagnosis of a severe childhood disease known as neuroblastoma (NB). HVA and VMA analyses are assayed by a number of different techniques, including the spot test, thin-layer chromatography, high-performance liquid chromatography, gas chromatography, mass spectrometry, and enzyme-linked immunoassay [6]. Currently, there is only limited knowledge on the diagnosis of NB using selective receptors in the field of electrochemical sensors. So far, the search for a suitable design of synthetic receptors to detect neuroblastoma markers and suitable approaches to their deposition on an electrode surface is a work in progress. The known receptors for the recognition of VMA/HVA include metaiodobenzylguanidine (mIBG) [7], α-cyclodextrin (α-CD) [8], and L-Leucine [9]. Protocols for the deposition of a receptor in the form of polymeric films derived from Tröger’s base [10], neutral red [11], and a cobalt bis(dicarbollide) derivative have been proposed [12]. For the diagnosis of neuroblastoma, the levels of VMA and HVA are determined in a urine sample with an average pH of about 6.0. An overview of non-modified and polymer-modified electrodes by Baluchova et al. [11] showed that a number of electrodes are applicable in the pH range 2.0–4.0. There is another important factor in the applicability of a sensor for the diagnosis of neuroblastoma, which is a metabolite concentration higher than 5.5 × 10^−5^ M. Therefore, there is still a demand for the introduction of new specific functionalized supramolecular systems capable of detecting NB metabolites at physiological pH and operating at concentrations above 5.5 × 10^−5^ M. A urea derivative was synthesized for the binding of VMA and HVA (Figure 1). In the design of a reported receptor, we attempted to meet two main criteria: (i) to maintain the selective group for carboxylate recognition and (ii) to incorporate a polymerizable group that will drive the receptor’s anchoring/attachment onto the surface of the electrode. Because both VMA and HVA include a carboxyl group, a receptor including urea groups, which are effective hydrogen bond donor sfor carboxylate recognition, should be one of the best candidates [13]. Furthermore, -NH-containing receptors such as acids could discriminate anions according to their basicity; the higher the basicity of the anion, the stronger the hydrogen-bonding interaction [13]. Anchoring/attachment of the urea derivative to the electrode surface should occur through the electrochemical oxidation of a thiophene group.

It was found that the electrochemical deposition of a supramolecular receptor through polymerisable units presumes the conservation of the main recognizing binding modes [10,12,14]. However, it is impossible to exclude the fact that the recognizing sites of a receptor deposited onto an electrode surface might interact with the electrode material and thus lose their capability to recognize the analyte of interest. The objective of this work was to compare the recognition capabilities of the urea moiety to discriminate structural analogues VMA and HVA on the surface of graphite and platinum electrodes. In this context, this work is a pilot investigation to examine the effect of the electrode surface material (platinum and graphite) on the discriminating ability of the urea moiety. It demonstrates the advantages of urea-derivative-modified graphite electrodes compared to non-modified ones at detecting VMA and HVA in a mixture.

## 2. Materials and Methods

### 2.1. Reagents

The derivative of urea was synthesized according to the procedure in [14], starting from 3-aminothiophene and methylene(diphenylene) diiosocyanate. The final product was obtained as a gray powder, with an 87% yield. Homovanillic acid (HVA, 4-hydroxy-3-metoxyphenylacetic acid) and vanillylmandelic acid (VMA, DL-4 hydroxy-3-methoxymandelic acid, 99%) were purchased from TCI (Zwijndrecht, Belgium) and Sigma-Aldrich (St. Louis, MI, USA), respectively. Tetrabutylammoniumtetrafluoroborate (TBABF_4_, 99%) was obtained from Sigma-Aldrich (USA). The inorganic reagents and organic solvents used were of analytical grade and did not require further purification unless otherwise specified (Lachema, Brno, Czech Republic). Double-distilled water was used throughout the experiment.

### 2.2. Density Functional Theory Calculations

Density functional theory (DFT) calculations were used to obtain a better insight into the analyte–receptor binding. The receptor and all the analytes were optimized at the B3LYP/6-31+G(d,p) level with the conductor-like polarizable continuum model (CPCM) to take into consideration implicit solvent effects. For each compound, the Boltzmann weights of the stable geometries were evaluated using the calculated enthalpies, and only the most abundant conformers were selected for further simulations of the analyte–receptor interactions. The calculated electrostatic potential of the receptor indicates that suitable binding sites for anions are located at the NH groups of the urea part of the receptor. For all four analytes, the most stable conformer was placed in the vicinity of the optimized receptor so that the negatively charged –COO^−^ groups were oriented towards the urea NH groups with the O···H distances of the OH being approximately 2 Å, and the resulting complexes were further optimized at the B3LYP/6-31 + G(d,p)/CPCM level. The interaction energies were calculated as follows:

$$E_{int}=E_{complex}-\left(E_{receptor}+E_{anion}\right).$$

### 2.3. FTIR Spectroscopic Characterization of Modified Electrode Surfaces

The FTIR spectra of the urea-derivative receptor electrochemically deposited on the surface of platinum (Pt/Receptor) and graphite (G/Receptor) screen-printed electrodes (ø = 3.1416 mm^2^) were recorded in the range of 4000-400 cm^−1^ with a Nicolet iS50 FTIR spectrometer (Thermo Fisher Scientific, Waltham, MA, USA) with a resolution of 4 cm^−1^ using a ZnSe crystal as the ATR accessory. A pure powdered receptor sample or Pt/C electrodes with the receptor immobilized on their surface were placed on an ATR crystal and pressed using a calibrated pressure tower. Two hundred and seventy-six scans were collected for each spectrum. All spectra were corrected for carbon dioxide and humidity in the optical path. ATR and baseline corrections of spectra were made using the software OMNIC 8.2 (Thermo Fisher Scientific, Waltham, MA, USA). Final figures were prepared in the software Origin 6.0 (Microcal Origin, Northampton, MA, USA).

### 2.4. Electrochemical Studies

All electrochemical experiments were performed with a Palmsens 3 (PalSmSens BV, Houten, The Netherlands). Electrochemical measurements were carried out with a three-electrode system, which has a platinum plate as the counter electrode, Ag/AgCl (3 M KCl) as the reference electrode, and platinum (Pt/Receptor) and graphite (G/Receptor) screen-printed electrodes (ø = 3.1416 mm^2^, BVT, Czech Republic) as the working electrodes.

The deposition of the receptor on the surface of G electrodes was carried out using electrochemical oxidation by a cyclic voltammetry technique (Figure S1 and Scheme S1). The polymerization mixture was 2 mM of receptor dissolved in the mixture (ACN/DMSO + 0.05 M TBABF_4_). Potential was scanned in the range from −0.2 to +1.9 V at a scan rate of 50 mV s ^−1^, 10 cycles.

Electrochemical impedance spectroscopy (EIS) experiments were carried out with a potential of 0 V, amplitude of 10 mV and in the frequency range of 50 to 10 mHz (69 points). The EIS signal of the modified electrodes (G/Receptor) was recorded in 5 mM K_3_[Fe(CN)_6_]: K_4_[Fe(CN)_6_] (1:1) with the addition of 0.05 M KCl. The experimental Nyquist plots were fitted in a Palmsens 3 (PalSmSens BV, Netherlands) with Zview software. To eliminate electrode-to-electrode signal variation in the evaluation of electrode sensitivity, the following equation was used:

*EIS signal* = ((*R*_n_ − *R*_0_)/*R*_n_) × 1000,

where *R*_0_ and *R*_n_ are the resistances to charge transfer (*R*_ct_) of the modified G/Receptor electrodes recorded in the supporting electrolyte before and after adding different concentrations of the tested analytes, respectively.

Differential pulse voltammetry (DPV) measurements were performed from 0.0 to 1.0 V with E_step_ = 5 mV, E_pulse_ = 50 mV, and scan rate 10 mV s^−1^. The dependence ΔI signal = f(log(c(analyte)) was found in a 0.1 M phosphate buffer with 0.14 mM NaCl added (PBS, pH 7.2), which was used for the electrochemical measurements. The determination of both VMA and HVA was carried out using a standard addition method in the model samples in the absence and presence of each structural analogue.

## 3. Results and Discussion

### 3.1. Density Functional Theory Calculations

**Figure 2:**
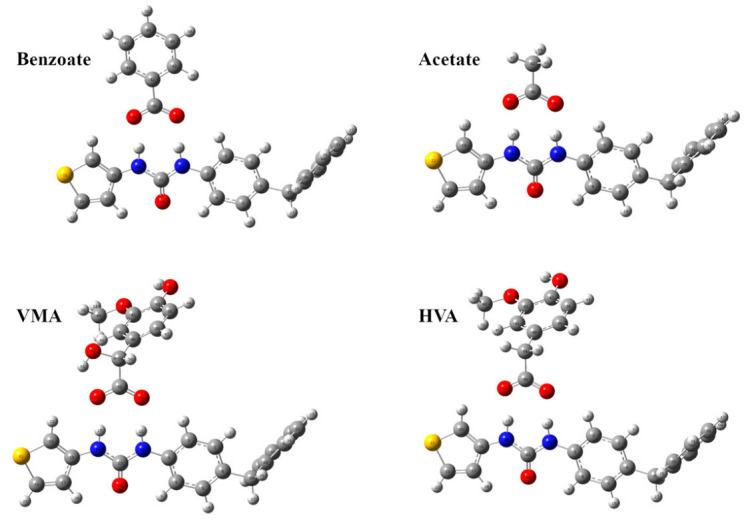
Optimized geometries (B3LYP/6-31+G(d,p)/CPCM) of analyte–receptor complexes.

The DFT calculations were used to characterize the nature of the analyte–receptor interactions. However, only the binding between the analytes and the monomeric form of the receptor in the solution was investigated, as the modeling of the oligomeric receptor bound onto the electrode surface would have been very time-consuming. The thorough conformational search of the receptor and analytes yielded stable geometries, and only the conformers with the highest Boltzmann weights (calculated using Gibbs free energies) were used for modeling mutual interactions. Two basic models were considered: the most likely interaction of –COO^−^ groups with the NH groups of the receptor’s urea moiety, and hydrogen bonding between the analytes’ OH groups (where present) and the receptor’s carbonyl group. However, the interactions between the analytes’ alcohol groups and the receptor’s carbonyl group were found to be disadvantageous due to steric hinderance. The calculated energies of the optimized complexes (Figure 2) were compared to those of the separate compounds (Table 1). The calculated interaction energies would indicate higher values of association constants than the experimentally derived values. However, the model is significantly simplified, and for instance solvent effects may play an important role in the analyte–receptor binding. More importantly, the calculated values reflect the experimental findings well when considering the relative values of association energies, showing that the affinity of VMA is slightly lower than that of HVA. This can be explained by the presence of an intramolecular hydrogen bond that is formed between an OH and carboxyl group (distance ~1.9 Å) that thus weakens the –COO^−^···HN interaction between the VMA and receptor.

### 3.2. FTIR Spectroscopy: Modified Electrode Surface Characterization

**Figure 3:**
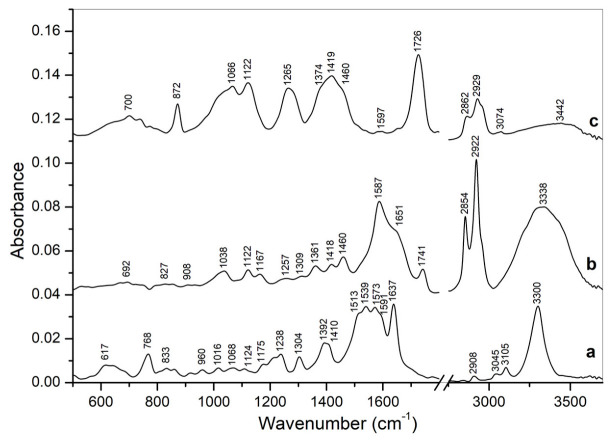
FTIR spectra of urea derivative before (**a**) and after electrochemical oxidation on the graphite (**b**) and platinum (**c**) screen-printed electrode surfaces.

The FTIR spectra confirmed the presence of an oxidized urea derivative on the surfaces of the modified platinum (Pt/Receptor) and graphite (G/Receptor) screen-printed electrodes (Figure 3). In addition, depending on the material of the electrodes, the molecules of the initial substance underwent significant reorganizational changes. The differences in the FTIR spectra of the studied samples reflect the transformations observed. There was an intense band at 3300 cm^−1^ assigned to NH stretching vibrations of urea moieties, and several weak bands at ~3105 cm^−1^ (=CH stretching in thiophene ring), ~3045 cm^−1^ (=CH stretching in benzene ring), and ~2908 cm^−1^ (CH_2_ stretching in alkyls) that were all observed in the high-wavenumber IR spectral region for the urea derivative (Figure 3a). A significant broadening and shift of the band at 3300 cm^−1^ to 3338/3442 cm^−1^ confirmed the polymerization of the urea derivative on the surfaces of the G electrodes (Figure 3b,c). The NH stretching band was intense for the surface coating of G electrodes, whereas with the Pt electrodes this band was significantly reduced. For the modified Pt electrode, the =CH stretching bands of the benzene and thiophene fragments in the urea derivative receptor only remained as a weak band at 3074 cm^−1^, but with the modified G electrode, these bands did not appear at all. The intense IR bands of asymmetric and symmetric CH_2_ stretching observed at 2922/2929 and 2854/2860 cm^−1^, respectively, arose from TBAFB_4_ used as a component of the polymerization mixture. This may also have contributed to the broadening of the bands at 1460 cm^−1^ (CH_2_ scissor) and 1361−1374 cm^−1^ (CH_3_ symmetric bending) with the modified electrodes [14]. These bands are absent in the FTIR spectrum of the starting material. The region of the overlapped C=O stretching, CN stretching, and NH bending bands showed significant changes. For the urea derivate, there was a band at 1637 cm^−1^ found in this region and assigned mainly to the C=O stretching vibrations. On the surfaces of modified C/Pt electrodes, this band shifted to 1741/1726 cm^−1^, which indicates a possible weakening of inter- or intramolecular hydrogen bonds with the participating C=O and NH groups, and the release of CO moieties from this interaction. However, for the G electrode, the band at 1741 cm^−1^ exhibited a significant decrease in intensity, while with the Pt-electrode, the band at 1726 cm^−1^ increased significantly. The bands of the para-disubstituted benzene ring at 1591 cm^−1^ (C=C stretching vibration), bands in the region 1290−900 cm^−1^ (in-plane C-H deformations) and 768 cm^−1^ (out-of-plane C-H deformations), and the thiophene ring vibrations at 1513 and 1392 cm^−1^ were significantly increased in intensity and broadened, but only for the polymer film on the surface of the Pt electrode. These bands were weak for the polymerized urea derivative on the surface of the G electrode, and there was a significant boost to the N-H bending vibration at 1573 and 1539 cm^−1^. In the polymer film, these vibrations appeared at 1651 and 1587 cm^−1^. These bands were absent for polymers on the surface of the Pt electrode. We assume that the described changes are associated with a different way of orienting the urea derivative molecules on the surface of the G and Pt electrodes after electrochemical oxidation. The weakening of the vibrations of CO, benzene, and thiophene moieties can indicate their orientation inside the polymer film on the surface of the G electrode. The enhancement of NH vibrations indicates their location at the outer layer of the G electrode surface. With the Pt electrode, the disappearance of the NH bands, the gain of the CO bands, and the increase in intensity and broadening of the benzene and thiophene bands can appear when the CO bonds, the benzene ring, and the thiophene ring are oriented towards the outside of the polymer film while the NH groups are oriented towards the inside.

### 3.3. Electrochemical Impedance Spectroscopy: Recognition at Electrode–Solution Interface

**Figure 4:**
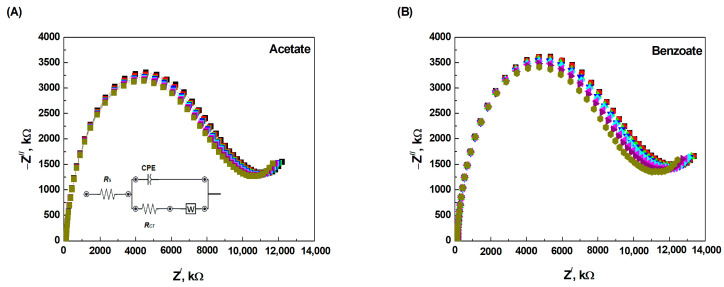

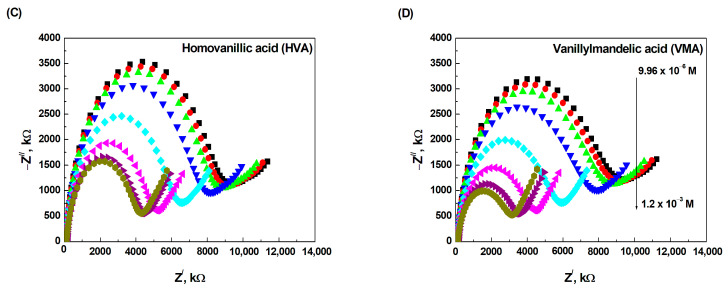
EIS spectra obtained with urea-derivative-modified graphite electrode toward tested carboxylate anions: acetate (**A**), benzoate (**B**), homovanillic acid (**C**), vanillylmandelic acid (**D**). Experimental conditions: aqueous solution of 5 mM K_3_[Fe(CN)_6_]: K_4_[Fe(CN)_6_] (1:1) with the addition of 0.05 M KCl. Concentrations: 9.96 × 10^−6^ M (black), 1.99 × 10^−5^ M (red), 3.99 × 10^−5^ M (green), 7.97 × 10^−5^ M (blue), 1.59 × 10^−4^ M (cyan), 3.16 × 10^−4^ M (magenta), 6.24 × 10^−4^ M (purple), 1.2 × 10^−3^ M (dark yellow). Inset (**A**) shows the Randles equivalent circuit model used for fitting all experimental EIS spectra, where *R*_s_ is the solution resistance, *R*_ct_ is the charge-transfer resistance, and CPE is the constant phase element.

The application of the receptor as the recognizing element for the analyte in question for an electrochemical sensor requires its anchoring onto an electrode surface. In this context, we were interested in determining and comparing the bulk and surface affinity for the urea receptor toward the chosen carboxylates. Electrochemical impedance spectroscopy (EIS) is an effective technique for the non-destructive monitoring of recognition processes that takes place at the electrode–solution interface [15,16]. The impedimetric signal is the result of the adsorption, diffusion of ions, and charge transfer of redox species. The binding of VMA/HVA affects the electron exchange between the modified electrode surface and the redox probe (5 mM K_3_[Fe(CN)_6_]: K_4_[Fe(CN)_6_] (1:1)), which leads to a change in its resistance to charge transfer (*R*_ct_). The *R*_ct_ is represented by the semicircle diameter on a Nyquist plot (Figure 4). The *R*_ct_ changes with increasing analyte concentration can be used to determine the surface affinity between the attached urea receptor and the chosen carboxylates in a quantifiable manner. The binding constants of the modified electrode surface with carboxylates were determined using the Langmuir adsorption isotherm. The properties of the G/Receptor electrode were compared with those of the Pt/Receptor electrode (Figure S2). For the G/Receptor electrode, the results of the EIS measurements are presented in Figure 4.

A decrease in impedance was observed with an increase in both the VMA and the HVA concentration, as evidenced by the decreasing height and diameter of the semicircle. The binding of the analyte onto the modified surface often leads to an increase in its resistance [17,18]. However, a less well-known phenomenon can also occur, in which the impedance of the modified surface is decreased after the binding of analytes [19,20,21,22,23]. The reason for such behavior may be heterogeneity effects, doping with anions, or a partial ionic exchange at the interface between the electrolyte and electrode surface. The morphology of the electrode material should be taken into account (Figure S3).

The comparison of the experimental values of association and adsorption constants (Table 2) leads to the following important conclusions: The recognition of carboxylates by the urea derivative is significantly affected by anchoring the urea moiety onto an electrode surface. The urea moiety is capable of interplay with the platinum surface and, consequently, of losing an expected affinity toward the carboxy group at the electrode–solution interface. Moreover, the basicity of the carboxy group is an important factor for discriminating VMA and HVA.

**Figure 5:**
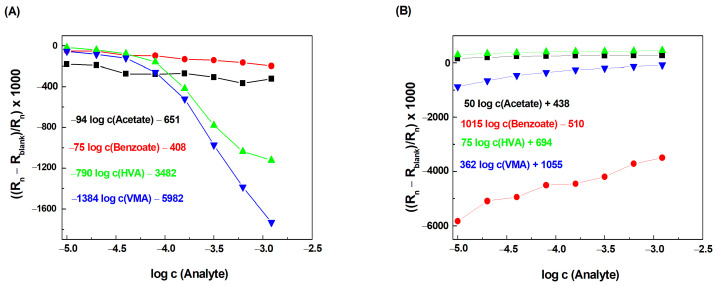
Comparison of EIS response obtained during EIS measurements with urea-derivative-modified graphite (**A**) and platinum (**B**) electrodes toward carboxylate anions. Experimental conditions: aqueous solution of 5 mM K_3_[Fe(CN)_6_]: K_4_[Fe(CN)_6_] (1:1) with the addition of 0.05 M KCl. Colors: acetate (black), benzoate (red), homovanillic acid (HVA, green), vanillylmandelic acid (VMA, blue).

**Scheme 1:**
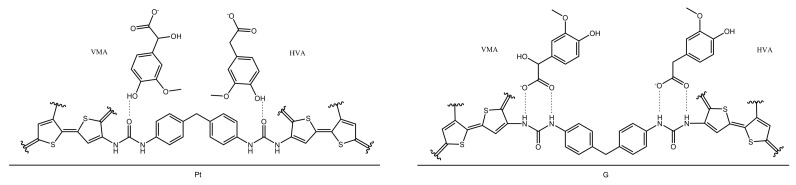
Interaction of HVA and VMA with platinum and graphite surface modified with electrochemically oxidized urea derivative.

In addition, the effect of the electrode material was obvious in the comparison of the sensitivity obtained for the Pt/and G/Receptor electrode to carboxylates (Figure 5). While resistance decreased with increasing concentration with a G electrode, the opposite trend (namely increasing resistance) was observed with a Pt electrode. Currently, we might take this phenomenon as confirmation of a different interaction mechanism between the urea derivative deposited on the Pt and G electrode (Scheme 1).

### 3.4. Differential Pulse Voltammetry: Detection of Metabolites

**Figure 6:**
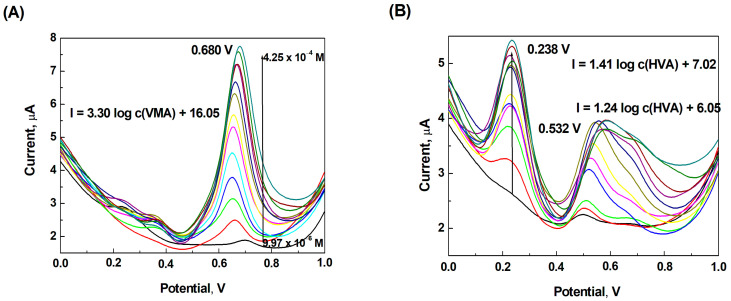
DP voltammograms of VMA (**A**) and HVA (**B**) recorded at G/Receptor modified electrode in phosphate-buffered saline at pH = 6. Concentrations: 9.97 × 10^−6^ M (black), 1.99 × 10^−5^ M (red), 3.99 × 10^−5^ M (green), 7.97 × 10^−5^ M (blue), 1.19 × 10^−4^ M (cyan), 1.58 × 10^−4^ M (magenta), 1.97 × 10^−4^ M (yellow), 2.35 × 10^−4^ M (dark yellow), 2.74 × 10^−4^ M (dark blue), 3.12 × 10^−4^ M (purple), 3.50 × 10^−4^ M (burgundy), 3.87 × 10^−4^ M (olive), 4.25 × 10^−4^ M (dark cyan).

The electrochemical behavior of VMA and HVA was studied on a G/Receptor modified electrode. The oxidation of the tested metabolites bonded onto the modified surface takes place at different potentials. A concentration dependence over the range 9.96 × 10^−6^–4.25 × 10^−4^ M was obtained at E_a_ = 0.680 V for VMA (Figure 6A), while two potentials, namely E_a1_ = 0.238 V (3.99 × 10^−5^–3.12 × 10^−4^ M) and E_a2_ = 0.532 V (9.97 × 10^−6^–2.35 × 10^−4^ M), were observed upon the oxidation of HVA (Figure 6B).

**Figure 7:**
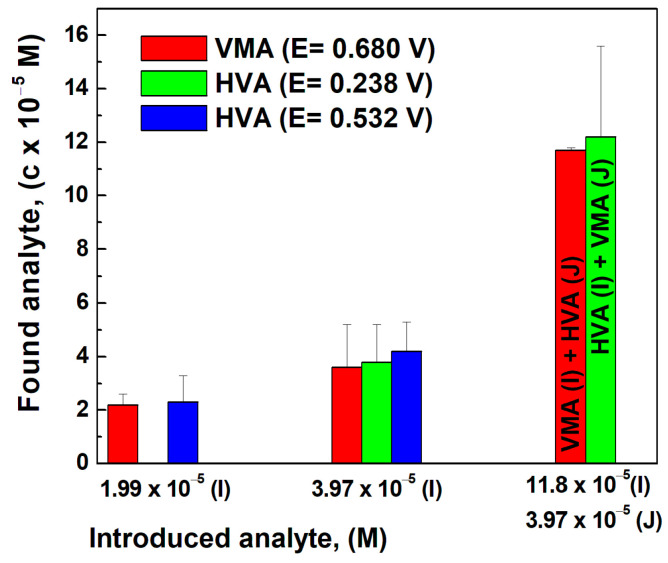
Detection of individual metabolites in the absence and presence of their structural analogue.

The experimental findings showed that (i) 1.99 × 10^−5^ M is the determined concentration for VMA (E_a_ = 0.680 V, S_r_ = 0.12, recovery 110%) and HVA (E_a2_ = 0.532 V (S_r_ = 0.28, recovery 115%) in model samples; (ii) the peak at E_a1_ = 238 V can be used for the quantification of HVA both in the absence and presence of a structural analogue from a concentration of 3.97 × 10^−5^ M; (iii) VMA has a greater interfering effect than HVA (Figure 7). The interfering effect of VMA could result from its high affinity to the urea moiety due to higher basicity, which is in agreement with experimental values of adsorption constants obtained from the EIS experiments (Table 2).

G/Receptor electrodes prepared using the same procedure are expected to give reproducible responses. Therefore, three replicates were prepared and compared. The G/Receptor electrodes exhibited acceptable reproducibility for

VMA: 9.96 × 10^−6^–1.58 × 10^−4^ M, E_a_ = 0.680 V (S_r_ = 0.10),

HVA: 9.96 × 10^−6^ − 4.25 × 10^−4^ M, E_a1_ = 0.238 V (S_r_ = 0.21) and E_a2_ = 0.532 V (S_r_ = 0.16).

**Figure 8:**
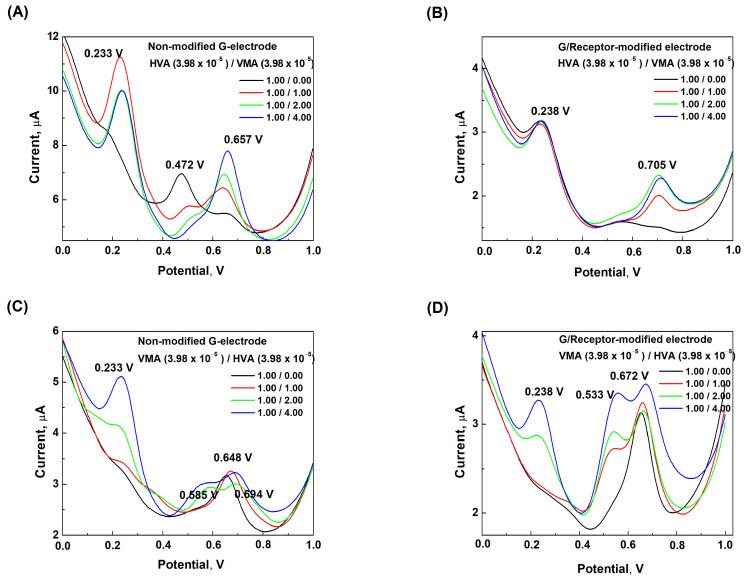
DP voltammograms for different ratios of HVA and VMA in mixture recorded at non-modified (**A**,**C**) and G/Receptor modified electrode (**B**,**D**) in samples of synthetic urine at pH = 6. For (**D**), the composition mixture is the same as for (**C**).

It should be taken into account that the non-modified electrode can have specificity towards HVA and VMA, and their specificity can be different for various forms of carbon. For example, MWCNTs/SPE, edge plane pyrolytic graphite electrodes, and anodically oxidized boron doped diamond exhibited 0.380 V and 0.430 V oxidation potential for VMA in 1 mM phosphate buffer, pH 6.85 [25]. Unfortunately, oxidation potentials for the tested metabolites are only known at acidic pH in the literature [11]. Urine is a matrix in which the detection of the level of neuroblastoma metabolites is conducted [26]. Concentrations higher than 5.5 × 10^−5^ M are dangerous and are used to diagnose neuroblastoma. Here, we would like to show the advantages of modifying the electrode surface. Therefore, DP voltammograms recorded for a mixture of metabolites with non- and modified electrodes have been compared in samples of synthetic urine (Figure 8). The individual HVA and VMA oxidation peaks are observable and separated (Figure 8B,D) at the G/Receptor electrode. These experiments demonstrate the selectivity of the designed sensor system based on the urea derivative.

The proposed sensor, which works over a range of both low and high concentrations of VMA/HVA, was compared with the sensor systems reported in the literature (Table 3).

The experimental findings show that the proposed electrode might also be developed for future medical applications.

## 4. Conclusions

The urea derivative was deposited onto the graphite and platinum electrode surfaces via its thiophene unit using electrochemical polymerization, characterized spectroscopically and tested by differential pulse voltammetry with structural analogues VMA and HVA. According to the DFT calculations, the interactions of all the analytes with the receptor are energetically favorable. The FTIR spectra reveal the polymerization of the urea derivative via its thiophene group and the different orientation of the urea moiety on a platinum and graphite surface. The availability of the urea moiety on the graphite surface for the carboxy group of the analyte of interest leads to the discrimination of VMA and HVA based on the difference in their basicity. The selectivity between metabolites and modified surfaces was confirmed and determined using EIS and DPV methods. The EIS selectivity was based on the change in resistance to charge transfer for the redox probe (K_3_[Fe(CN)_6_]: K_4_[Fe(CN)_6_]) as a result of the adsorption: VMA (K_Ads_ = (5.18 ± 1.95) × 10^5^) and HVA (K_Ads_ = (4.78 ± 1.58) × 10^4^). The DPV selectivity was based on monitoring the characteristic potential corresponding to the oxidation of the analyte bound to the urea-derivative-modified surface: VMA (E_a_ = 0.680 V); HVA (E_a1_ = 0.238 V and E_a2_ = 0.532 V). In contrast to the non- and modified electrodes reported in the literature, the proposed G/Receptor electrode enables detecting the above-mentioned metabolites of neuroblastoma within a concentration range with diagnostic significance. This concept could be further extended for future medical applications.

## Figures and Tables

**Table 1:** Calculated interaction energies (B3LYP/6-31+G(d,p)/CPCM) and corresponding values of association constants for analyte–receptor systems.

Analyte	ΔE (kJ/mol)	*K*_Ass_ 1:1
Acetate	−56.4	7.6 × 10^9^
Benzoate	−55.3	4.9 × 10^9^
VMA	−48.6	3.0 × 10^8^
HVA	−55.2	4.7 × 10^9^

**Table 2:** Comparison of surface to bulk affinity of the urea derivative to selected carboxylates (n ≤ 5).

Analyte	pK_a_/pK_b_	Receptor ^(a)^		G/Receptor	Pt/Receptor
		*K*_Ass_ 1:1	β_Ass_ 1:2	*K*_Ads_	*K*_Ads_
Acetate	4.756/9.244	1.97 × 10^3 (c)^	9.65 × 10^5^	(5.32 ± 2.99) × 10^5^	(3.86 ± 1.61) × 10^5^
Benzoate	4.2/9.8	1.01 × 10^3 (b)^	2.53 × 10^5^	(1.3 ± 0.4) × 10^6^	(5.30 ± 3.77) × 10^5^
VMA	3.4/10.6	4.20 × 10^2 (b)^	4.41 × 10^4^	(5.18 ± 1.95) × 10^5^	(1.84 ± 0.61) × 10^5^
HVA	4.4/9.6	1.72 × 10^3 (b)^	7.40 × 10^5^	(4.78 ± 1.58) × 10^4^	(2.76 ± 0.77) × 10^5^

^(a)^ The data are from reference [14]; ^(b)^ 1H NMR titration in DMSO-d6, ^(c)^ UV-Vis titration in DMSO; pK_a_ = 12.6 for acetic acid in DMSO; pK_a_ = 11.1 for benzoic acid in DMSO [24].

**Table 3:** Detection of VMA and HVA using differential pulse voltammetry—comparison of our results with those published elsewhere.

Analyte	Polymer-Layer/Electrode	Detected Concentration, μM	Supporting Electrolyte	References
VMA	Tröger’s Base/G	40–100 10–100	0.1 M PB, pH = 7.0	[10]
	Nafion/GCE ^1^	8–100	0.1 M PB ^2^, pH = 3.0	[11]
	Poly(neutral red)/GCE	4–10 20–100	0.1 M PB ^2^, pH = 3.0	[11]
	Urea-derivative/G	9.96–158	0.1 M PBS, pH = 6.0	This work
HVA	l-Leucine/Sol-Gel-C	0.4–100	0.1 M PBS, pH = 4.0	[9]
	Tröger’s Base/G	40–100		[10]
	Nafion/GCE ^1^	2–10 10–100	0.1 M PB ^2^, pH = 3.0	[11]
	Poly(neutral red)/GCE	4–100	0.1 M PB ^2^, pH = 3.0	[11]
	Urea-derivative/G	9.96–624	0.1 M PBS, pH = 6.0	This work

^1^ GCE—glassy carbon electrode. ^2^ PB—phosphate buffer.

## Data Availability

Not applicable.

## Supplementary Materials

The following supporting information can be downloaded at: https://www.mdpi.com/article/10.3390/s23073727/s1, Figure S1: Cyclic voltammogram obtained during electrochemical oxidation of urea derivative on the surface of the G electrode; Figure S2: Comparison of EIS response obtained during EIS measurements with urea-derivative-modified platinum electrode towards tested carboxylate anions. Experimental conditions: aqueous solution of 5 mM K_3_[Fe(CN)_6_]: K_4_[Fe(CN)_6_] (1:1) with the addition of 0.05 M KCl; Figure S3: SEM electron micrographs (magnification 10,000×) of graphite electrode before (A) and after modification with the urea-derivative; Scheme S1: Thiophene derivative oxidation.
