# Supplementary material for: Voltammetric Detection of Vanillylmandelic Acid and Homovanillic Acid Using Urea-Derivative-Modified Graphite Electrode
Source: Sensors (Basel). 2023 Apr 4;23(7):3727. doi: 10.3390/s23073727 (PMC10098763; doi:10.3390/s23073727)
Supplement: Supplementary file 1 [file sensors-23-03727-s001.zip › sensors-2251849-supplementary.pdf]

Voltammetric Detection of Vanillylmandelic acid and Homovanillic acid using Urea – derivative modified Graphite Electrode

Tatiana V. Shishkanova [1], František Králík [1], Alla Synytsya [1]
[1] University of Chemistry and Technology Prague, Technická 5, 16628 Prague 6, Czech Republic, E-mail:tatiana.shishkanova@vscht.cz

Cyclic voltammetry: deposition of urea-derivative on the electrode surface

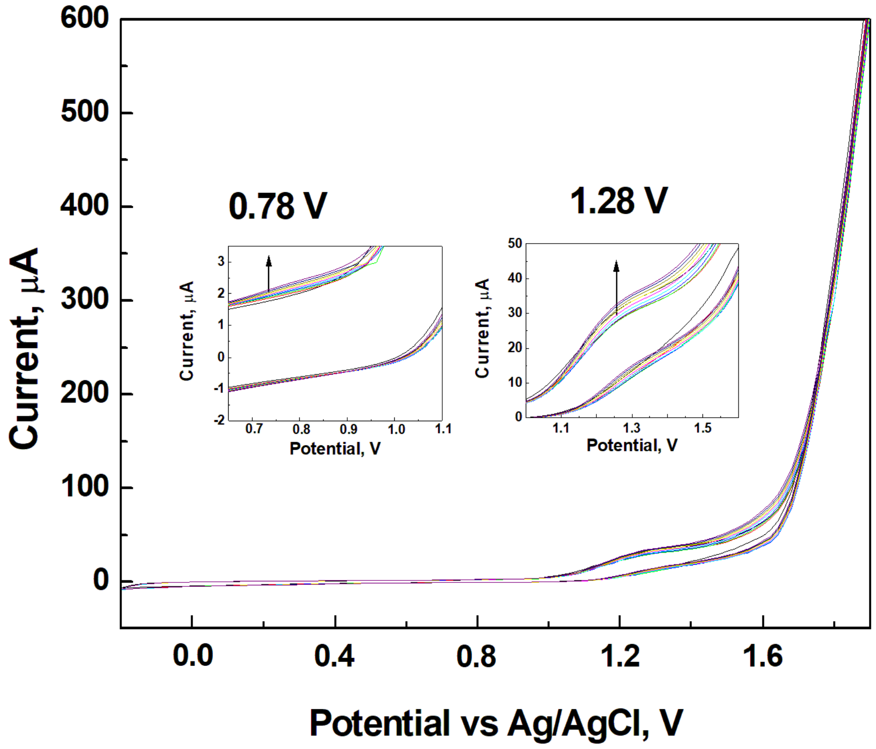

Figure S1. Cyclic voltammogram obtained during electrochemical oxidation of urea-derivative on the surface of the G electrode.

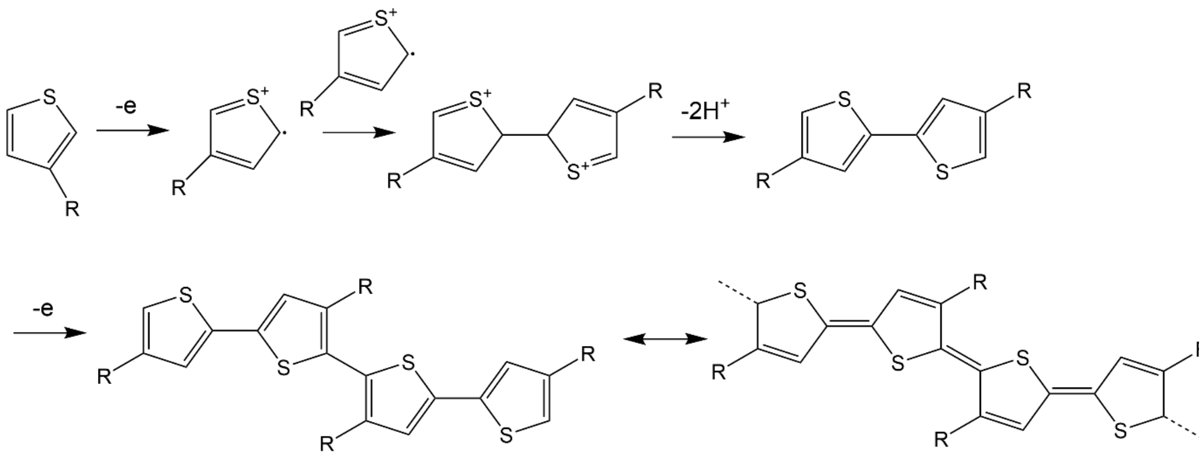

Schema S1. Thiophene derivative oxidation

Electrochemical impedance spectroscopy: Recognition on electrode-solution interface

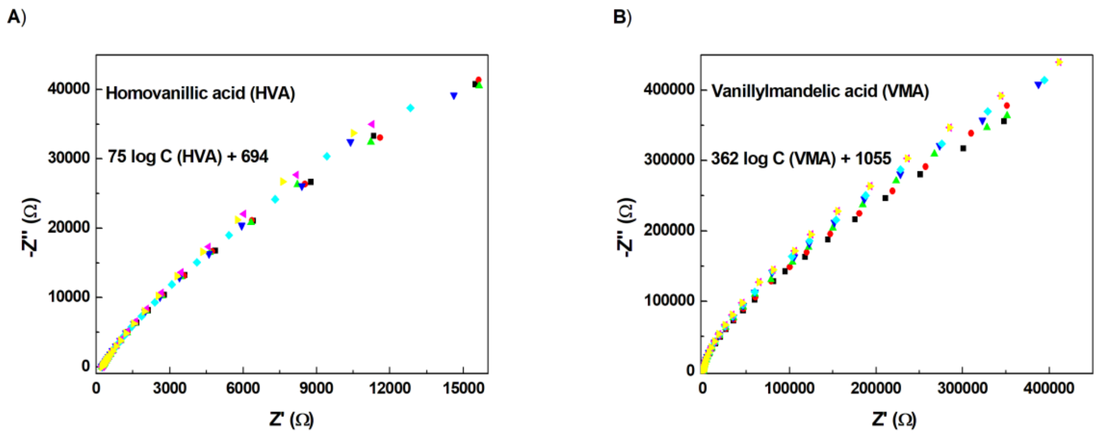

Figure S2 The EIS response for urea-derivative modified platinum electrode towards structural analogues. Experimental conditions are in chapter „*2.4. Electrochemical studies*“.

Scanning electron microscopy characterization

The morphology of electrode surfaces was studied with Mira 3 LMH (Tescan) field emission scanning electron microscope at accelerating voltage 5 kV.

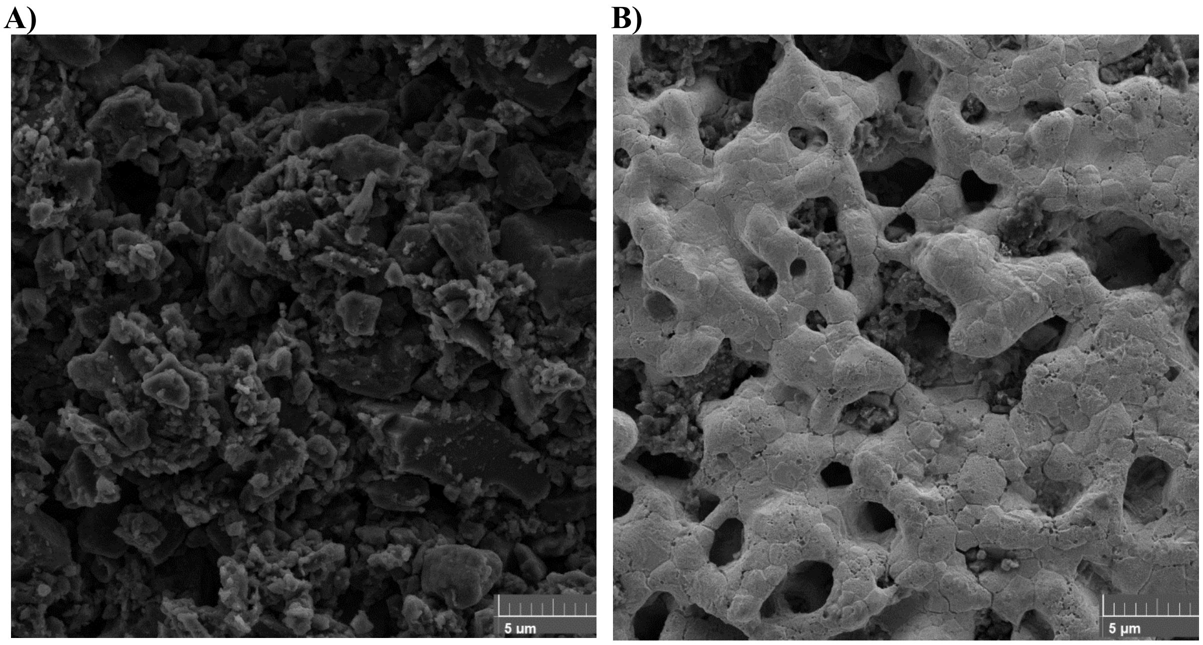

Figure S3. SEM electron micrographs (magnification 10 000x) of graphite electrode before (A) and after modification with the urea-derivative.
